# Severity of Dental Caries and Saliva Properties in Diabetes Mellitus

**DOI:** 10.3390/dj13120553

**Published:** 2025-11-24

**Authors:** Ashwaq Alkahtani, Aylin Baysan

**Affiliations:** 1 Department of Dental Health, The College of Applied Medical Sciences (CAMS), King Saud University, Riyadh 11433, Saudi Arabia; asalkahtani@ksu.edu.sa; 2 Institute of Dentistry, Bart’s and the London Faculty of Medicine and Dentistry, Queen Mary University of London, London E1 2AD, UK

**Keywords:** dental caries, diabetes mellitus type 2, salivary properties, international caries detection and assessment system (ICDAS)

## Abstract

Background/Objective: Diabetes mellitus type 2 (T2DM) is a significant global public health concern. This analytical cross-sectional study aimed to evaluate the impact of T2DM on the severity of dental caries and salivary properties. Methods: A total of 182 participants (n = 91 *per* group) were recruited into the T2DM and non-diabetes (ND) groups. Unstimulated whole saliva (UWS) and blood samples for HbA1c were collected. Clinical assessments included plaque index, SoproLIFE, ICDAS, and severity index for root caries. Results: A total of 92 male and 89 female participants were recruited in this study. The results showed that the mean of UWS pH was slightly lower in the T2DM (6.65 ± 1.12) than the ND group (6.88 ± 0.49), however the difference was statistically insignificant (*p* = 0.065). The mean saliva buffer capacity was almost identical between the two groups, with values of 7.38 ± 3.2 in T2DM and 7.37 ± 2.83 in the ND groups (*p* = 0.973), indicating an insignificant difference. The mean saliva spinnbarkeit was slightly high and insignificant in the T2DM (4.57 ± 4.34) compared to the ND group (3.69 ± 3.6) (*p* = 0.141). The T2DM group had a significantly high proportion of participants with very low saliva flow rate (<0.1 mL/min) in comparison to ND (20.9% vs. 5.5%) (*p* = 0.004). ICDAS scores were significantly higher in T2DM (0.67 ± 0.32) when compared to the ND group (0.57 ± 0.24) (*p* = 0.014). ICDAS scores 3 and 6 were significantly higher in T2DM (2.76 ± 2.66; 0.86 ± 2.61) in comparison to ND (2.10 ± 2.00, 0.26 ± 0.96). Conclusions: Within the limitations of this analytical cross-sectional study, participants with T2DM demonstrated a higher risk of developing severe carious lesions and exhibited low salivary pH and flow rate when compared with non-diabetic individuals, supporting a possible association between glycemic status, salivary alterations, and caries severity.

## 1. Introduction

The intricate relationship between oral health and systemic conditions has become an increasingly prominent focus in the fields of dentistry and medicine. Among the various systemic disorders, the surge in type 2 diabetes mellitus (T2DM) worldwide has prompted an increased interest in understanding its potential impact on oral health [1], particularly its association with dental caries. With the rise in T2DM prevalence globally, there is increasing evidence regarding the multifaceted interactions between diabetes and oral health. This emphasizes the necessity for a comprehensive approach to patient care [2]. 

Dental caries, a widespread chronic disease that impacts individuals across the globe, is intricately connected to risk factors including lifestyle choices, dietary habits, and overall systemic health [3]. Emerging evidence points toward a potentially high susceptibility of individuals with T2DM to develop dental caries [4,5]. This association underscores the need for further exploration and a consideration of the interplay between diabetes and oral health, especially for dental caries.

Saliva plays a key role in maintaining oral homeostasis [6]. This complex fluid regulates pH, remineralizes enamel and dentine, and provides antimicrobial defence. Therefore, changes in the quantity and quality of saliva in individuals with T2DM may contribute to an increased susceptibility to dental caries [7,8].

The association between type 2 diabetes mellitus (T2DM) and dental caries is both clinically significant and biologically intriguing. T2DM, characterized by systemic metabolic dysregulation, elevates blood glucose levels and impairs insulin function, potentially influencing the composition of saliva and the oral microbiome [9]. These alterations may create an environment conducive to dental caries development, further exacerbated by the compromised immune responses observed among individuals with T2DM [10].

Caries-prone individuals often exhibit reduced salivary flow rates, lower pH values, and diminished mineral content, all of which disrupt the balance between demineralization and remineralization processes [11]. However, despite the biological plausibility of this link, the current evidence regarding the association between T2DM and dental caries remains inconclusive. Many previous clinical studies were limited by small sample sizes and cross-sectional designs, which restrict the ability to draw robust conclusions [12]. Some investigations have reported higher numbers of decayed, missing, or filled teeth or surfaces (DMFT or DMFS indices) among diabetic patients compared with non-diabetic individuals [7,13,14], whereas others found no significant differences [15,16,17].

Furthermore, only a few studies have explored the influence of glycemic control on dental caries, yielding scattered and inconsistent findings from small-scale clinical cohorts [14,18]. Interestingly, a population-based analysis from the Korean National Health and Nutrition Examination Survey revealed a significantly higher prevalence of untreated dental caries among individuals with uncontrolled diabetes compared with metabolically healthy participants [19].

Although the present study also employs a cross-sectional design, the reasons were to address limitations of current evidence by including an adequately powered and balanced sample of diabetic and non-diabetic participants, employing standardized ICDAS scoring, ensuring examiner calibration, and incorporating comprehensive salivary analyses. While causality cannot be inferred, this design provided important comparative insights into the relationship between T2DM, salivary characteristics, and dental caries severity. Consequently, there is a need for well-designed, large-scale epidemiological and longitudinal cohort studies, using validated caries assessment tools, to strengthen the evidence base regarding the association between T2DM and dental caries.

## 2. Materials and Methods

### 2.1. Sample Size

An overall sample size of 91 individuals (182 in total) was assessed for the extent of dental caries between the groups with a power of 80% and a significance level of 0.05. This calculation assumed that 50% and 30% of individuals in the study’s groups, respectively, would have carious lesions, representing the minimum clinically relevant difference. The required sample size was estimated using G*Power version 3.1.9.7 (Heinrich Heine University, Düsseldorf, Germany).

### 2.2. Study Participants

This comparative cross-sectional observational study was conducted between December 2020 and November 2021. Prior to the study, ethical approval was obtained from the Office of Research Ethics Committees (REC reference number: 08/H0702/54, Approval date: 21 February 2020). A total of 400 patients were initially screened, and 182 participants (n = 91 per group) were recruited for this analytical cross-sectional study according to inclusion and exclusion criteria (Appendix A Table A1) using multiple approaches and were requested to sign the written consent forms.

Participants were recruited from various clinical settings within NHS Hospitals, including various Outreach Centres and Rheumatoid, General Endocrinology, Metabolic Medicine, and Diabetes clinics, as well as through the Diabetes database at the NHS Hospital.

There were different recruitment approaches. Approximately 75% were recruited via local media advertisements and public engagement, while 15.3% were recruited from the diabetes database through invitation letters.

The study included two groups: (1) adults with a previously confirmed medical diagnosis of type 2 diabetes mellitus (T2DM group) and (2) adults with no known history of diabetes (non-diabetic group, ND group). Diabetic participants were identified through documented medical records or physician confirmation, while non-diabetic individuals were recruited from the same community population and reported no history of diabetes or anti-diabetic medication use.

### 2.3. Data Collection

The principal investigator (PI), a trained dental practitioner, conducted data collection in two phases:

- Phase one involved the collection of saliva and blood samples for the analysis of saliva properties and HbA1c levels.
- Phase two comprised a clinical examination, which included assessments of plaque index and coronal and root caries severity.

All data collection was carried out at the Dental Hospital, equipped with standard dental chairs and overhead halogen lighting. The same setting was used for both saliva sampling and clinical examinations to maintain consistency. During the caries assessment, each tooth surface was cleaned and air-dried for approximately five seconds with an oil-free three-way syringe, in accordance with ICDAS guidelines, to ensure optimal visibility and accuracy. Infection-control protocols and aseptic procedures were followed throughout the assessments.

The duration of type 2 diabetes mellitus (T2DM) was obtained from participants’ medical history. For analysis, it was grouped as ‘≤15 years’ or ‘>15 years’ since diagnosis to distinguish recent from long-standing cases.

### 2.4. Saliva Samples

Unstimulated whole saliva (USWS) was collected to measure the salivary flow rate, pH, buffer capacity, and spinnbarkeit. Those who agreed to take part in the study were advised to refrain from eating, drinking, and smoking for at least one to two hours prior to the assessment appointment [20]. To reduce the circadian effect, the test was conducted between 9:00 a.m. and 3:30 p.m. [20,21]. The dental chair was set up for the participants, who were instructed to sit comfortably and tilt their heads slightly forward. USWS was collected by instructing participants to swallow first and then drool any saliva that collected in the floor of the mouth into a pre-weighed plastic cup for 5 minutes. After five minutes, the saliva vial was reweighed and recorded in milliliters per minute (mL/min), assuming that 1 g/min equals 1 mL/min [21]. The ‘saliva-check buffer testing mat’ kit (GC Dental Products Corp.,Tokyo, Japan) was used to determine the pH and buffering capacity.

The Neva Meter device (IMI—021, Ishikawa Ironworks Co., Ltd., Niigata, Japan) was used to measure the spinnbarkeit of saliva. The measurement was conducted five times per sample to ensure accuracy and minimize variability. To refine the data, the highest and lowest readings were excluded, and the average of the remaining three measurements was calculated. This approach, based on the methodology described by Gohara et al. [22], ensures reliable and reproducible results by mitigating the effects of outliers.

All saliva samples were collected by the principal investigator (PI), a qualified dental practitioner, who received hands-on training from the saliva-testing kit manufacturers prior to data collection to ensure a standardized collection protocol. In addition, the PI previously gained experience in salivary analysis through assisting in a PhD-level clinical research project in a separate clinical setting, which provided extensive exposure to saliva handling and measurement protocols. All samples were collected under identical environmental conditions to maintain consistency, accuracy, and reliability throughout the study.

### 2.5. Clinical Assessments

Plaque accumulation was evaluated using the Silness and Löe Plaque Index (PI) (Appendix A Table A2) during clinical examination. Each tooth surface was scored from 0 to 3 based on plaque presence, and a mean PI score was calculated for each participant. Dental caries assessments were conducted after supragingival and subgingival debridement, when/if required, and included polishing with a non-fluoridated prophylaxis paste (NUPRO Dentsply, Bensheim, Germany). All tooth surfaces, when coronal/root caries were present, were evaluated according to ICDAS [23] and the root severity index [24,25,26]. In addition, a light-induced fluorescence evaluator (SoproLIFE©, Acteon group, La Ciotat, France) was utilized to detect dental carious lesions.

Root caries severity was assessed using the Root Caries Severity Index (RCSI) proposed by Baysan and Lynch [25], which classifies lesions according to surface texture, cavitation, and accessibility for plaque removal (Appendix A Table A3). The scoring ranged from Score 0 (sound dentine) to Score IV (soft structure), allowing detailed differentiation between active and inactive lesions. This index builds upon the validated clinical criteria of Beighton et al. [24], which emphasize lesion hardness as a key indicator of activity. Including the RCSI provided a clear, reproducible method to grade the leathery surface lesions into clinically meaningful severity categories.

SoproLIFE^®^ (Acteon group, La Ciotat, France) was used in blue fluorescence mode as a supplementary diagnostic aid alongside ICDAS visual scoring to support the detection of early or uncertain lesions. Fluorescence images were captured and evaluated according to the criteria (Appendix A Table A4) described by Rechmann et al. [27]. The mean fluorescence score for each participant was derived from all examined teeth to complement ICDAS assessments and enhance diagnostic accuracy.

The researchers had been previously calibrated in ICDAS, ICCMS, and SoproLIFE fluorescence scoring during a preliminary laboratory-based study assessing the diagnostic performance of SoproLIFE for early caries detection [28]. This calibration demonstrated moderate inter- and intra-examiner agreement (κ = 0.52 and 0.58, respectively), confirming the validity and reproducibility of the scoring procedure. In addition, both investigators received hands-on demonstration and training from the SoproLIFE manufacturer on device operation, fluorescence interpretation, and image recording before initiating the clinical phase. The same investigators performed all ICDAS and SoproLIFE examinations in the present study to maintain reliability, accuracy, and internal validity across participants.

### 2.6. Blood Test for HbA1c Analysis

All participants, including both type 2 diabetes mellitus (T2DM) and non-diabetic (ND) groups, underwent HbA1c testing to confirm glycemic status and ensure accurate group classification. For the HbA1c measurements, the Quo-Test^®^ HbA1c Analyzer (EKF-diagnostic GmbH, Leipzig, Germany), a fully automated point-of-care (POC) analyzer, was used. This device employs patented boronate affinity fluorescence quenching technology to measure glycated hemoglobin levels from a 4 μL sample obtained via finger prick.

The process started with the collection of a blood droplet, ensuring the participant’s finger was warm, dry, clean, and free from any interfering substances. A single-use lancet was used for the puncture, and a droplet of blood about the same width as the blood collector was obtained. The blood droplet was then transferred to the test cartridge using a blood collector and placed into the free cavity on top of the cartridge. Subsequently, the test cartridge, along with the blood collector, was inserted into the test chamber of the analyser within one minute of the blood sample collection to prevent result inaccuracies.

The Quo-Test analyzer is CE-marked, indicating compliance with European Union regulations for medical devices, ensuring its quality and safety for monitoring HbA1c levels in diabetes patients. Analysis using the Quo-Test analyzer provides the results within four minutes, and the outcome is reported in the new International Federation of Clinical Chemistry (IFCC) standard units. These steps were carried out systematically according to the manufacturer’s instructions to ensure the accuracy and efficiency of each sample analysis.

The principal investigator (PI) received an in-person demonstration from the Quo-Test^®^ manufacturer prior to the start of data collection to ensure accurate and standardized operation of the device. This training covered proper finger-prick technique, sample handling, and cartridge insertion to minimize pre-analytical variability and ensure consistent measurements across all participants.

### 2.7. Statistical Analyses

The categorical data were numerically coded to be statistically analysed. Results are presented as mean ± SD and percentage. Statistical significances and differences between T2DM and ND values were evaluated by using either parametric or non-parametric tests according to data distribution. Independent-sample t-tests were applied for continuous variables that were normally distributed, while the chi-square test was used for categorical variables. One-way analysis of variance (ANOVA) was also employed to compare the means of quantitative variables between groups. *p*-values of <0.05 were considered as significant. Pearson’s correlation analysis was performed to measure the linear association between HbA1c and salivary spinnbarkeit and between salivary pH and ICDAS scores. All statistical analyses were carried out by using Statistical Package for Social Science, Version 21.0 (SPSS, IBM Corp., Armonk, NY, USA).

## 3. Results

### 3.1. Participant Characteristics

The mean age was 61 ± 11.7 for the T2DM and 51 ± 11.2 in the ND group. The majority of the T2DM participants were males at 67%, whereas 33% were female participants, while the ND group had 64.8% females and 35.2% males. Of the T2DM participants, 38.5% were Asian British, while the second most common ethnicity was White/Caucasian with 28.6%. Black/African British participants contributed 17.6%, becoming the third most common in the T2DM group. For the ND group, 41.9% of the participants were self-identified as other ethnicities without any details provided, while the second most common ethnicity was Black/African British with 28.6%. White/Caucasian participants contributed 16.5%, becoming the third most common in the ND group. Lastly, 12.1% of the participants were Asian British. Comprehensive socioeconomic and behavioral characteristics of the same participant cohort, including education, income, employment status, and plaque control factors, were previously reported in detail [29].

### 3.2. Salivary Parameters

Table 1 illustrates the difference with regard to salivary properties, i.e., flow rate, pH, buffer capacity and spinnbarkeit between groups. Three T2DM participants failed to provide sufficient unstimulated saliva. The mean of saliva pH was slightly lower in the T2DM group (6.65 ± 1.12) when compared to the ND group (6.88 ± 0.49), although the difference was statistically insignificant (*p* = 0.065). Similarly, the mean saliva buffer capacity was almost identical between the two groups, with values of 7.38 ± 3.2 in the T2DM group and 7.37 ± 2.83 in the ND group (*p* = 0.973), indicating an insignificant difference. The mean saliva spinnbarkeit was slightly high in the T2DM group (4.57 ± 4.34) when compared to the ND group (3.69 ± 3.6). However, the difference was statistically insignificant (*p* = 0.141).

Individuals with T2DM were more likely to have a very low or low saliva flow rate when compared to those without diabetes. The distribution of salivary flow rate categories was significantly different between the T2DM and ND groups (overall Chi-square test, *p* = 0.004). Notably, a higher proportion of T2DM participants exhibited a very low saliva flow rate (<0.1 mL/min) compared to ND (20.9% vs. 5.5%). Differences in other categories, such as low and normal saliva flow rates, did not reach statistical significance in pairwise comparisons (low: 35.2% vs. 31.9%, *p* = 0.525; normal: 44.0% vs. 62.6%, *p* = 0.025). The significant *p*-value refers only to the overall distribution and not to individual categories.

### 3.3. Salivary pH Levels

In the T2DM group, there were 6 (6.7%) participants with acidic saliva (pH 5.0–5.8), while a total of 40 (44.9%) participants had moderately acidic saliva (pH 6.0–6.6), and 43 (48.3%) participants presented with neutral saliva (pH 6.8–7.8). This difference in the proportion was statistically significant (*p* < 0.05).

In the ND group, 7 participants (7.7%) had high acidic saliva (pH 5.0–5.8), while a total of 32 (35.2%) had moderate acidic saliva (pH 6.0–6.6), and 52 participants (57.1%) had neutral saliva (pH 6.8–7.8). This difference in the proportion was statistically significant (*p* < 0.05). However, when the comparison between T2DM and ND was carried out to assess the salivary pH, the difference in the proportion was statistically insignificant (*p* > 0.05; Table 1).

### 3.4. Buffer Capacity

In the T2DM group, there were 24 individuals (27.27%) with a neutral buffer capacity level and 39 (44.32%) and 25 (28.41%) with low and very low buffer capacity levels, respectively.

In the ND group, there were 17 individuals (18.7%) with a neutral buffer capacity level and 24 (26.4%) and 50 (54.9%) with low and very low buffer capacity levels, respectively. The difference in T2DM was statistically insignificant (*p* > 0.05). However, there were statistically significant differences in the ND group (*p* < 0.05; Table 1).

### 3.5. Dental Caries and Plaque Status

Table 2 presents the dental status of the study participants with regard to data on the number of total teeth, sound, treated and untreated teeth, intra-coronal and extra-coronal restorations (e.g., crowns, onlays, inlays), unreplaced missing teeth, and missing teeth. The results are displayed as mean, median, and standard deviation, along with *p*-values indicating statistical significance. Although all participants retained several sound coronal teeth, none of the individuals in either group were completely free of coronal caries. Each participant presented with at least one treated or untreated coronal lesion.

The T2DM group exhibited slightly lower mean values across most parameters compared to the ND group, although these differences were statistically insignificant. However, the T2DM group showed significantly high mean values for unreplaced missing teeth (*p* < 0.05).

The mean plaque index (PI) was slightly higher in the T2DM group (0.87 ± 0.44) than in the non-diabetic group (0.79 ± 0.49), but this difference was statistically insignificant (*p* = 0.272).

### 3.6. Dental Caries Severity Scores

Table 3 displays the distribution of carious lesions based on ICDAS scores and the overall mean of caries based on the ICDAS between the groups. The results indicated that the mean scores for carious lesions scored 3 and 6 were significantly high in the T2DM group (*p* < 0.05), while there was no significant difference for other ICDAS scores. The overall ICDAS scores were significantly high in the T2DM.

### 3.7. Categories According to the Severity of Coronal Caries

The carious lesions were classified according to their severity as initial (ICDAS scores 1 and 2), moderate (ICDAS 3 and 4), and severe lesions (ICDAS 6 and 7). These scores were then compared with the glycemic control and duration of DM within T2DM, as shown in Table 4. There was no significant difference in the distribution of carious lesions between the controlled and the uncontrolled and the duration of DM, but participants with slightly high moderate and extensive lesions presented with uncontrolled and 15+ years of diabetes history when compared to controlled and <15 years of diabetes history.

### 3.8. Severity of Root Caries

A total of eleven T2DM participants presented with 30 root carious lesions, while only seven participants in the ND group had a total of eight root caries (*p* < 0.05). Sixty percent of T2DM root lesions scored with severity indices of 1, 2, and 3, while only twenty-five percent of ND root lesions had an SI score of 2 (*p* = 0.072) (Appendix A Table A5).

### 3.9. Correlation Analysis

A low positive correlation was observed between salivary pH and ICDAS scores within the T2DM group, indicating that lower salivary pH was associated with greater caries severity. A further low positive correlation was found between HbA1c and salivary spinnbarkeit (r = 0.26, *p* = 0.016). These were the only significant correlations identified in the study.

## 4. Discussion

This analytical cross-sectional study aimed to investigate the association between type 2 diabetes mellitus (T2DM) and the severity of dental caries (coronal and root), and to evaluate the relationship between key risk factors, including metabolic status (HbA1c) and salivary properties (pH, flow rate, buffering capacity, and spinnbarkeit). This study is unique in its comprehensive approach, integrating standardized diagnostic tools (ICDAS and SoproLIFE) with detailed salivary analyses to assess caries patterns in individuals with and without T2DM.

Different avenues were approached to recruit 182 participants. Approximately 75% of study participants were recruited through public engagement and advertisement in local media; this could be attributed to the fact that recruitment took place after the COVID-19 national lockdown, which was accompanied by recommendations to suspend elective medical and dental care. Therefore, the demand for dental examinations was high, and many study participants were willing to accept the invitation to participate in this research in exchange for free dental care. In addition, the positive response rate from the letters that were sent to T2DM participants through the local diabetes database was only 15.3%. This could be related to the fact that an individual with diabetes who considered themselves to be at a high risk of COVID-19 infection due to the nature of the disease might have been reluctant to attend the dental appointment during the pandemic.

The findings align with previous reports that males account for a large share of the diabetic population, suggesting a significant gender misdistribution. In regions such as the Middle East and North Africa, diabetes is more commonly diagnosed in middle-aged men in comparison to women [30]. According to the results of the US National Health and Nutrition Examination Survey, men are at a higher risk of developing diabetes than women [31]. British Asian participants constituted the largest ethnic group of people with T2DM in this study. The high prevalence of diabetes in Asia has previously been documented [32]. Multiple factors, including ethnicity, race, and genetics, have been linked to the development of T2DM [33]. Age, obesity, family history of diabetes, sedentary lifestyle, and the prevalence of other modern lifestyle disorders like hypertension and dyslipidaemia were linked to an increased risk of developing diabetes [34].

The saliva properties that were evaluated in the study were saliva pH, buffer capacity, flow rate, and spinnbarkeit. The T2DM group showed a slightly lower salivary pH when compared with the non-diabetic group, consistent with the previous findings [35]. The findings of this study support the hypothesis that there is a potential association between the rate of dental caries in participants and their salivary pH levels. In this analytical cross-sectional study, a significant but weak positive correlation was observed between salivary pH and ICDAS scores within the T2DM group, indicating that low salivary pH was associated with an increased number of severe dental caries. These results can be attributed to the impact of pH levels on bacterial growth and the diffusion of acid through the pellicle, which leads to demineralisation of hard tooth tissue structures [36].

The normal range for whole saliva flow rates during rest is above 0.25 mL/min. It should be noted that the occurrence of hyposalivation symptoms is observed at much lower rates, ranging from 0.10 to 0.01 mL/min [37]. In this cross-sectional study, the prevalence of decreased salivary flow rate was higher in the T2DM group. The resting salivary flow rate was categorized as very low, low, and normal, with prevalence rates of 20.9%, 35.2%, and 44%, respectively, in the T2DM group compared to the ND group (5.5%, 31.9%, and 62.6%, respectively). Previous studies indicated that uncontrolled diabetes can cause a decrease in salivary flow [37,38,39]. This decrease may be related to dehydration resulting from the excretion of excess glucose through the kidneys. In hyperglycemic states, dehydration may occur as the body attempts to restore glucose homeostasis. All participants were instructed to have an early breakfast and abstain from food for at least an hour before the saliva collection, so that the participants could be hyperglycemic at the time of sample collection. Although a diurnal pattern of salivary flow is recognized, this is unlikely to have affected the results since most of the samples were collected in the morning at similar times [40,41]. Any inconsistencies in the timing of sampling would be expected to be evenly distributed among all groups. Moreover, a considerable number of participants were taking medications known to decrease salivary flow, primarily as a result of their diabetic condition. According to Meurman et al. [42], the reduced salivary flow observed in T2DM may also be associated with the medications used in its management.

The autonomic nervous system regulates salivary secretion, and it is possible that the changes observed in salivary secretion in diabetic patients are due to diabetic autonomic neuropathy. Despite this, studies reported that there were no differences in the resting and stimulated rates of salivary secretion between patients with T2DM and the ND groups [42,43]. In this current study, there was no statistically significant correlation between saliva flow rate and overall ICDAS scores. In addition, there were insignificant differences between the two groups with regard to buffering capacity.

The spinnbarkeit represents one of the extensional rheological properties of saliva and reflects the ability to adhere to oral tissues, providing protective functions and enhancing lubrication [44]. Any alteration in this property may impair the adherence of saliva within the oral cavity, potentially leading to dryness and complications associated with xerostomia, such as difficulties in wearing dentures [45]. The spinnbarkeit of ND group presented with low values in this study when compared to the previous study, where the values were 28.54 ± 5.66 [45]. Interestingly, the T2DM group demonstrated a greater number of samples with spinnbarkeit above 10 mm (n = 11) when compared to the ND group (n = 5). This finding suggests that unstimulated saliva, regardless of diabetic status, may not significantly affect the Spinnbarkeit properties of saliva. Interestingly, there was a low positive correlation between the levels of HbA1c and the spinnbarkeit (r = 0.26, *p* = 0.016). Although there is limited explanation for this correlation in the literature, it can be speculated that both parameters are influenced by diabetes-related changes in salivary composition, which may ultimately alter spinnbarkeit. No association was found between spinnbarkeit and other salivary properties in this study.

The lack of a significant difference in plaque index between T2DM and non-diabetic participants indicates that variations in caries severity are unlikely to be explained solely by plaque accumulation. This aligns with previous research suggesting that metabolic control and salivary composition, rather than plaque levels alone, play a more prominent role in influencing caries susceptibility among individuals with diabetes [46].

A relationship between dental caries and Type 2 diabetes has a reasonable biological basis. However, clinical and epidemiological evidence supporting this association remains limited and somewhat inconsistent, as previous studies have produced conflicting results. In the present study, the prevalence of dental caries was assessed using the ICDAS criteria. ICDAS is currently considered the recommended standard for caries detection in clinical and research settings, as it allows classification of lesions along a continuum of severity. The use of ICDAS in this study enabled a detailed evaluation of carious lesion severity in both groups and the assessment of lesion distribution by code. The integration of SOPROLIFE imaging for early lesion detection further enhanced diagnostic accuracy and allowed for a more precise evaluation of enamel and dentine caries.

Analysis of the total mean ICDAS scores revealed that the overall severity of dental caries differed between the study groups. Participants with T2DM showed higher mean ICDAS values, indicating a greater extent of carious lesions compared with the ND group. Furthermore, the T2DM group exhibited a higher frequency of advanced lesions, particularly those classified as ICDAS codes 3 (localized enamel breakdown without visible dentine) and 6 (extensive cavities with visible dentine). When the lesions were categorized according to the ICCMS, participants in the T2DM group demonstrated a slightly higher prevalence of moderate and severe lesions compared to those in the ND group. These results are consistent with previous research by Abayon et al. [47], which reported moderate to severe dental caries among diabetic patients. The correlation between the ICDAS scores of the diabetic and those of the non-diabetic populations indicated that diabetes patients tended to score high in the severe category [48]. However, Lalla et al. [49] failed to detect any differences in American diabetic participants with regard to dental caries severity.

Dietary behavior plays a key role in the occurrence and severity of dental caries [50], even among individuals adhering to dietary restrictions for T2DM management. Although diabetic diets typically limit refined sugar intake, the pattern and frequency of carbohydrate consumption i.e., frequent small meals aimed at stabilising blood glucose may still sustain acidogenic bacterial activity and reduce oral pH [46]. In addition, chronic hyperglycemia may indirectly alter the oral environment through changes in salivary composition and buffering capacity, thereby amplifying the cariogenic potential of even modest carbohydrate exposure [51]. The observed differences in caries severity between the T2DM and non-diabetic (ND) groups in this study may therefore reflect a combined effect of metabolic dysregulation and subtle dietary variations, rather than high sugar consumption alone.

The prevalence of root caries has been steadily increasing in the UK, particularly among older adults [52]. Epidemiological data indicate that nearly one-third of adults over the age of 65 had root caries as early as 1998, while a later survey reported that 7% of adults over 16 had active root caries, rising to 20% in those aged 74 and older [53]. Although evidence directly linking diabetes to root caries remains limited, high occurrence of the root carious lesions among individuals with diabetes was reported [54]. Sharma et al. [54] demonstrated that 46% of individuals with diabetes had root caries, compared to 28.5% of non-diabetic individuals.. The present study similarly reported a high occurrence of root caries among participants with T2DM, highlighting the potential interplay between metabolic and oral factors. While there are biologically plausible explanations for this associatiob i.e., loss of attachment leading to gingival recession and root exposure to the oral environment, commonly seen in periodontitis. However, further investigation is required to establish any possible causality [55]. Within this context, exposed root surfaces may act as vulnerable sites for caries initiation and progression, particularly when accompanied by compromised salivary conditions or poor glycemic control.

Glycated hemoglobin (HbA1c) was used in this study as an established clinical biomarker for glycemic control. HbA1c reflects the average plasma glucose concentration over the preceding 8–12 weeks, thus providing a reliable long-term indicator of metabolic status. Elevated HbA1c levels (≥53 mmol/mol) have been consistently associated with oral complications, including increased caries activity, periodontal inflammation, and salivary dysfunction [56]. By using HbA1c, participants could be objectively stratified into controlled and uncontrolled diabetes subgroups, allowing meaningful comparison of salivary characteristics with glycemic control. This approach minimizes the influence of short-term glucose fluctuations and provides a more stable indication of metabolic control, strengthening the interpretation of the systemic–oral relationships observed in this study.

There is still limited evidence regarding the overall relationship between T2DM and oral health outcomes. One contributing factor to inconsistent findings across studies is the inadequate control of confounding variables such as socioeconomic status and dietary habits. In addition, few investigations have specifically focused on root caries in diabetic populations. Consistent with previous research [57], the current study found a high prevalence and activity of root caries among T2DM participants when compared to their non-diabetic counterparts, underscoring the need for targeted preventive strategies in this group.

These findings suggested that even though root caries was reported in ND participants, the severity and activity of root carious lesions were high in those with T2DM. Specifically, the reduced salivary pH and buffering capacity, together with the lower flow rate in T2DM participants, may have limited remineralization potential and promoted lesion activity on the exposed root surface. Although the differences in severity levels were not all statistically significant, the overall trend indicates that poor glycemic control and compromised salivary function could act synergistically to increase caries activity rather than mere prevalence. This aligns with the current study’s correlation findings between HbA1c and salivary spinnbarkeit, reinforcing the possible interplay between systemic metabolic state and salivary rheology in influencing lesion behavior.

These outcomes are in line with previous evidence. In this respect, Soni et al. [58], demonstrated that individuals with diabetes may be up to 42% more prone to developing root carious lesions, with a mean RCI score of 3.8 ± 11.6. A recent systematic review showed that individuals with T2DM were three times more likely to develop root caries than the ND group without DM [59], yet the present study extends this understanding by combining clinical caries assessment with detailed salivary analysis in the same cohort, providing a more integrated interpretation of the underlying association.

This study has certain limitations that should be considered. Although the sample size was adequate for the statistical analyses performed, it may restrict the generalizability of the findings. Future investigations should aim for larger, multi-center cohorts to enhance external validity and represent different population groups. Longitudinal studies are also warranted to confirm the temporal relationships suggested by the present cross-sectional findings. Further research incorporating stratified analyses by demographic variables and salivary biomarkers could provide deeper insight into the factors underlying the observed differences in oral health between T2DM and non-diabetic groups.

## 5. Conclusions

This analytical cross-sectional study identified a clear association between type 2 diabetes mellitus (T2DM) and the severity of dental carious lesions, particularly root surface involvement. Participants with T2DM exhibited a higher occurrence and activity of both coronal and root caries compared with non-diabetic individuals. These findings suggest that glycemic status and salivary characteristics may play contributory roles in caries progression among diabetic populations. Given the study’s cross-sectional design, causal relationships cannot be inferred; however, the observed associations highlight the clinical importance of integrating oral health evaluation into diabetes management. Regular oral health assessment, salivary monitoring, and patient education on oral self-care should form part of comprehensive diabetes care to reduce the burden of dental caries in this population. Further longitudinal and multi-center studies are recommended to confirm these associations and explore underlying mechanisms.

## Figures and Tables

**Table 1:** Salivary parameters (pH, buffering capacity, spinnbarkeit, flow rate of saliva) in the T2DM and ND groups.

Saliva Parameters	T2DM (n = 88)	ND (n = 91)	*p* Value
Average Salivary Parameters			
Saliva pH (Mean ± SD)	6.65 (±1.12)	6.88 (±0.49)	0.065
Saliva Buffer Capacity (Mean ± SD)	7.38 (±3.20)	7.37 (±2.83)	0.973
Saliva Spinnbarkeit (Mean ± SD, mm)	4.57 (±4.34)	3.69 (±3.60)	0.086
Spinnbarkeit above 10 mm, n (%)	11 (12.5%)	11 (12.5%) <0.001	
Spinnbarkeit below 10 mm, n (%)	77 (87.5%)	77 (87.5%)	
Flow Rate Categories			
Very low (<0.1 mL/min), n (%)	16 (18.1)	6 (5.5)	0.004 *
Low (0.1–0.25 mL/min), n (%)	32 (36.3)	29 (31.9)	
Normal (>0.25 mL/min), n (%)	40 (45.4)	56 (62.6)	
Total	88 (100)	91 (100)	
Buffer capacity levels			
Neutral, n (%)	24 (27.27)	17 (18.7)	0.283
Low, n (%)	39 (44.32)	50 (54.9)	
Very low, n (%)	25 (28.41)	24 (27.4)	
Saliva pH Level			
High acidic, n (%)	6 (6.7)	7 (7.7)	0.407
Moderate acidic, n (%)	40 (44.9)	32 (35.2)	
Neutral, n (%)	43 (48.3)	52 (57.1)	

Independent-sample *t*-test used for continuous variables (Mean ± SD); Chi-square test applied for categorical variables (n, %).* Indicates statistical significance at *p* < 0.005.

**Table 2:** Dental status of the study participants.

	T2DM (n = 91)			ND (n = 91)			
	Mean	Median	SD±	Mean	Median	SD±	*p* Value
**Total number of teeth**	23.8	24	6.05	25.57	26	5.34	0.04 *
**Sound teeth**	9.54	10	4.28	10.53	11	4.24	0.12
**Number of teeth with dental caries**	14.27	14	4.65	15.03	15	3.83	0.23
**Number of restoration surfaces**	8.53	6	8.76	8.28	5	8.67	0.44
**Extra-coronal restoration (crowns, onlays, inlays)**	1.03	0	1.84	1.24	0	1.83	0.44
**Unreplaced missing teeth**	6.70	5	5.33	4.54	4	4.15	0.001 *
**Replaced missing teeth**	6.45	5	5.35	8.18	7	6.05	0.043 *

Independent-sample *t*-test used for continuous variables (Mean ± SD). * A *p* value < 0.05 was considered statistically significant.

**Table 3:** The caries pattern distribution as per the ICDAS score between groups.

ICDAS Score		Groups		
		T2DM	ND	
		Mean ± SD	Mean ± SD	*p* Value *
0	Sound tooth surface	102.0 ± 24.89	114.6 ± 21.82	<0.001 *
1	Initial caries	1.10 ± 2.67	1.44 ± 2.70	0.393
2	Distinct visual change in enamel	9.81 ± 5.40	9.97 ± 5.07	0.843
3	Localized enamel breakdown due to caries with no visible dentine	2.79 ± 2.66	2.10 ± 2.00	0.049 *
4	Underlying dark shadow from dentine	1.07 ± 4.76	0.47 ± 1.09	0.248
5	Distinct cavity with visible dentine	0.24 ± 0.82	0.11 ± 0.35	0.160
6	Extensive distinct cavity with visible dentine	0.86 ± 2.61	0.26 ± 0.92	0.042 *
	Overall ICDAS lesions	0.67 ± 0.32	0.57 ± 0.24	0.014 *
*p* value *		<0.00	<0.00	

Independent-sample *t*-test and Mann–Whitney U test used as appropriate. * A *p* value < 0.05 was considered statistically significant.

**Table 4:** Comparison of severity of dental caries with the glycemic control level and duration of DM within T2DM.

	Glycemic Control Mean ± SD			Duration of Diabetes Mean ± SD		
Severity of Dental Caries	Uncontrolled Above 48 mmol/L (n = 61, 62.74 ± 13.52)	Controlled Below 48 mmol/L (n = 30, 42.77 ± 3.11)	*p* Value	>15 Years	<15 Years	*p* Value
ICDAS mean	0.58 ± 0.25	0.53 ± 0.22	0.319	0.57 ± 0.26	0.57 ± 0.21	0.890
Initial	10.6 ± 6.28	11.5 ± 5.90	0.526	9.84 ± 4.85	12.28 ± 7.34	0.061
Moderate	4.14 ± 7.01	3.26 ± 3.13	0.514	4.02 ± 7.37	3.65 ± 3.69	0.773
Extensive	1.13 ± 2.85	0.96 ± 2.48	0.740	1. 20 ± 2.90	0.98 ± 2.38	0.703

Independent-sample *t*-test/Mann–Whitney U test used as appropriate (*p* < 0.05 considered significant).

## Data Availability

The original contributions presented in this study are included in the article. Further inquiries can be directed to the corresponding author.

## Appendix A

**Table A1:** Inclusion and Exclusion Criteria.

Inclusion Criteria	Exclusion Criteria
Participants who are male or female ≥ 18 years of age. For the test group, they have been diagnosed with type 2 Diabetes For the ND group, participants who are not diagnosed with type 2 Diabetes Participants having minimum of six natural tooth They are capable of giving informed consent They have the ability to understand and speak English They are able and willing to comply with all trial requirements They are not participating in another dental trial They are not diagnosed for cognitive defect due to mental illness, depression, Alzheimer’s disease, or dementia. No antibiotics, no steroidal and/or non-steroidal anti-inflammatory medication used during the last 3 weeks Participants who are not pregnant and also not breastfeeding Participations who are not in another dental study testing different dental products during the previous three months and during the study period	Participants who are edentulous Cognitive defect due to mental illness, depression, Alzheimer’s disease, or dementia. The presence of any hard or soft tissue tumours in the oral cavity Patients undergoing chemo and/or radiation therapy Any other significant disease or disorder which, in the opinion of the Investigator, may either put the participants at risk because of participation in the trial, or may influence the result of the trial, or the participant’s ability to participate in the trial. Any condition, which in the opinion of the investigator, would preclude participation by the subject (such as cross-infection control risk) Participants who are prescribed long-term systematic antibiotics Participants who are pregnant and breastfeeding Participations who are in another dental study testing different dental products during the previous three months and during the study period Participants who had additional fluoride treatment in the past 6/3 months Participants who are prescribed to use high fluoridated toothpaste

**Table A2:** Silness-Löe plaque index score criteria.

Score	Criteria
**0**	No plaque
**1**	A film of plaque adhering to the free gingival margin and adjacent area of the tooth, which cannot be seen with the naked eye. But only by using disclosing solution or by using probe.
**2**	Moderate accumulation of deposits within the gingival pocket, on the gingival margin and/ or adjacent tooth surface, which can be seen with the naked eye.
**3**	Abundance of soft matter within the gingival pocket and/or on the tooth and gingival margin.

**Table A3:** Severity Index of root caries.

Score	Texture	Description
0	Hard lesions	Hard structure as normal adjacent sound dentine
1	Leathery’ lesions (leathery—hard)	Easily cleansable and approaching a ‘Hard’ texture
2	Leathery’ lesions	Shallow, and where the surface of the exposed sound dentine could easily be maintained plaque-free
3	Leathery’ lesions (leathery—soft)	Difficult to maintain plaque-free, and large, cavitated ‘leathery’ lesions where pulpal integrity is judged to be at risk
4	Soft lesions	Soft structure

**Table A4:** Description of fluorescence images according to Rechmann et al. (2012) [27].

Score	Description	
**0**	Fissure appears as shiny green; enamel appears sound. A graphite-pencil-coloured thin shine/line is rarely observed	
**1**	Tiny, thin red shimmer in the pit and fissure system is viewed. No red dots appeared.	
**2**	In addition to tiny, thin red shimmer in pits and fissures possibly coming up the slopes darker red spots confined to the fissure are visible. There was no surface roughness.	
**3**	Dark red extended areas are confined to the fissures. Slight roughness is possible	
**4**	Dark red areas are wider than fissures. Surface roughness occurs. Possibly grey or rough grey zone may be visible	
**5**	Obvious enamel breakdown with visible bright red or dark dentine was observed.	

**Table A5:** Number of participants with root caries and the difference between the two groups at lesion level.

Variables	T2DM	ND	*p* Value
Distribution of root caries (n)	30	8	<0.00
Numbers of participants with root caries	11 (12.1%)	7 (7.7%)	<0.00
Root caries SI n (%)			0.072
SI: 0	12 (40%)	6 (75%)	
SI: 1	8 (27%)	2 (25%)	
SI: 2	4 (13%)	0 (0%)	
SI: 3	6 (20%)	0 (0%)	
SI: 4	0 (0%)	0 (0%)	
